# Immune Control of Herpesvirus Infection in Molluscs

**DOI:** 10.3390/pathogens9080618

**Published:** 2020-07-29

**Authors:** Jacinta R Agius, Serge Corbeil, Karla J Helbig

**Affiliations:** 1 School of Life Sciences, La Trobe University, Melbourne, VIC 3086, Australia; 19318704@students.latrobe.edu.au; 2 CSIRO, Australian Centre for Disease Preparedness, Geelong, VIC 3220, Australia; s.corbeil@csiro.au

**Keywords:** abalone, oyster, herpesvirus, mollusc, immune priming

## Abstract

Molluscan herpesviruses that are capable of infecting economically important species of abalone and oysters have caused significant losses in production due to the high mortality rate of infected animals. Current methods in preventing and controlling herpesviruses in the aquacultural industry are based around biosecurity measures which are impractical and do not contain the virus as farms source their water from oceans. Due to the lack of an adaptive immune system in molluscs, vaccine related therapies are not a viable option; therefore, a novel preventative strategy known as immune priming was recently explored. Immune priming has been shown to provide direct protection in oysters from Ostreid herpesvirus-1, as well as to their progeny through trans-generational immune priming. The mechanisms of these processes are not completely understood, however advancements in the characterisation of the oyster immune response has assisted in formulating potential hypotheses. Limited literature has explored the immune response of abalone infected with Haliotid herpesvirus as well as the potential for immune priming in these species, therefore, more research is required in this area to determine whether this is a practical solution for control of molluscan herpesviruses in an aquaculture setting.

## 1. Introduction

Several molluscan species, such as the pacific oyster (*Crassostrea gigas*, of the class Bivalvia) and the abalone (of the class Gastropoda), are of economic and ecological importance worldwide. Due to an increasing demand for their meat, they make up a large portion of the production in the aquacultural sector [1,2,3,4]. The main cultivated species of abalone in Australia include the blacklip, greenlip and hybrids of these two species [2,5]. As of 2019, approximately 55% of worldwide wild-caught abalone was produced in Australia. However, future growth in abalone production is expected as a result of an increase in aquaculture, emphasising the significance of these species in both a farmed and wild setting [1,2]. Several diseases exist that can disrupt production in these settings and it is estimated that US $2 billion is lost in revenue from diseases affecting farmed molluscs worldwide on a yearly basis [6]. Viral pathogens are one of the biggest threats to the shellfish industry and the diseases they cause are responsible for a large portion of these losses [6]. Herpesviruses are a family of large enveloped DNA viruses that are major viral pathogens affecting humans, animals and molluscs alike [3,7,8]. Ostreid herpesvirus (OsHV-1) and its genotypes, as well as Haliotid herpesvirus (HaHV-1), which infect oysters and abalone, respectively, have been associated with several outbreaks worldwide, including in Australia [9,10,11,12,13,14,15,16].

Biosecurity measures are inadequate in controlling such pathogens as evidenced by the losses both to the aquaculture industry and to native abalone species in the wild during these outbreaks, reinforcing the need for alternate strategies when dealing with herpesviruses. Recent research into the timing of oyster spat immersion has demonstrated that altering farming practices to obtain optimal water temperatures may alter the susceptibility of oysters to OsHV-1, however whether this work is translatable in practice to areas outside of the Ebro Delta region of Spain where the study was conducted remains to be seen [17]. Additionally, the usefulness of a temperature dependent approach during abalone spawning remains to be tested, and this approach may also not be useful in the case of an unexpected outbreak. Currently no vaccines or drugs exist to combat herpesvirus infection in molluscs, and detection of the virus in outbreaks often takes place after clinical signs are visible, limiting the practicality of enforcing movement restrictions in farmed settings [18]. This review aims to introduce the anti-viral immune response of molluscs and explore the feasibility of the new strategy, immune priming, in these animals.

## 2. Herpesvirus Infection in Molluscs

Serious economic losses have been attributed to viruses of molluscs, particularly herpesviruses that have significantly affected abalone and oysters in the wild and in aquacultural settings [14,16,19,20]. Herpes-like viral infection in an oyster was first described in the Eastern Oyster in 1972 (*Crassostrea virginica*), with viral imaging studies revealing a distinctive icosahedral structure characteristic of herpesviruses [21,22]. However, subsequent sequencing analysis revealed that OsHV-1 was only distantly related to other vertebrate herpesviruses and therefore represented a novel class of this virus family [21]. *Malacoherpesviridae* currently contains only two herpesvirus species [23,24]: Ostreid herpesvirus (OsHV-1) and Haliotid herpesvirus (HaHV-1) that mainly infect oysters and abalone, respectively. HaHV-1 was first discovered during a mass mortality event in *Haliotis diversicolor supertexta* in Tawain in 2003, with a subsequent outbreak of a similar virus seen in Australia in 2005 in both the black and green lip abalone (*Haliotis rubra* and *Haliotis laevigata*, respectively) [14,15]. However, more recent studies back-dated its occurrence to 1999 in Southern China populations of *Haliotis diversicolor supertexta* [25,26]. Many studies have now demonstrated that there are multiple OsHV-1 and HaHV-1 variants, which are generally geographically distributed, however recent sequence comparison analysis of both OsHV-1 and HaHV-1 variants revealed that although they appear to have common ancestors, they only share approximately 50% protein homology, making them very divergent viruses [12,14,15,27,28,29,30,31,32,33].

Variants of OsHV-1 have also been linked to events of mass mortality in other species of bivalves, including the blood ark clam *Scapharca broughtonii* and the farer’s scallop *Chlamys farreri,* highlighting the capability of this virus to infect several organisms from the class Bivalvia [31,34,35]. Additional reservoir hosts of OsHV-1 could potentially exist due to the detection of viral DNA in other species of bivalves, although further research is needed to characterise if the virus is truly replicative or just due to contamination from infectious water sources [36]. OsHV-1 and its associated variants were first linked to events of mass mortality in the Pacific Oyster (*Crassostrea gigas*) in France from 1993 to 2008, but since then have also been associated with disease in other countries including Australia [12,16,33,37]. HaHV-1 on the other hand was first recorded in 2003 in Taiwan and later in 2005 in Australia [23,26], where both outbreaks were associated with an acute onset of disease and high mortality [15,26].

HaHV-1, which is the etiological agent of the disease abalone viral ganglioneuritis (AVG) and OsHV-1 have a death rate close to 100% in infected animals [3,28]. AVG is characterised by its neuropathological effects, with the virus infecting the cerebral, pleuropedal, branchial and buccal ganglia, ultimately resulting in tissue necrosis and lesions [14,15,28,38,39]. As the nerve system is the primary target of the virus, the main clinical signs include poor pedal adhesion and swelling with prolapse of the mouth, which is not evident in a healthy animal [14]. These initial clinical signs eventually lead to the death of the animal in as short a period as 4–5 days following virus exposure [40].

In oysters, OsHV-1 cannot be classified by any characteristic symptoms or gross pathology other than non-specific signs, such as gaping shells or after an event of mass mortality [41]. OsHV-1 targets the connective tissues of organs and in particular, those of the gills and mantle [41]. Fibroblast-like cells, which make up these connective tissues, exhibit enlarged nuclei and chromatin in the perinuclear space leading to necrosis [3,42]. However, although the pathology of OsHV-1 and HaHV-1 differ, their ability to inflict acute widespread mortality is a common feature.

Due to the limitations of viral propagation and characterisation of OsHV-1 and HaHV-1, along with the absence of a continuous molluscan cell line, limited literature exists defining their lifecycles, however, the process has been described for vertebrate herpesvirus [43,44,45]. Herpesviruses are known to attach to host cells via a multitude of viral glycoproteins and host cell receptors [44,45]. Upon attachment, viral fusion then follows, whereby the virion envelope and the host cell membrane fuse. This leads to the release of the viral capsid and virus replication is initiated once the herpesvirus virion translocates into the nucleus [44]. Recently it has been inferred that the open reading frame 25 (ORF25) encodes viral membrane proteins involved in these first interactions between OsHV-1 and the host cell, however the identity of the host cell receptors still remains elusive, although integrins have been hypothethised to potentially play a role [46,47]. This is a first step into understanding the pathogenesis and viral life cycles of OsHV-1 but also highlights that we lack a thorough understanding of the pathogenesis of both OsHV-1 and HaHV-1.

## 3. Anti-Viral Immunity

The molluscan anti-viral immune response is not as well understood in comparison to other model invertebrate systems, such as the Fruit fly (*Drosophila*) and the mosquito (*Anopheles*) [7,48,49,50]. This poses a considerable risk for effective management strategies when novel viruses emerge. Although an abundance of literature exists on vertebrate immune systems, the confounding difference when compared to invertebrate systems is the absence of an adaptive branch of immunity and therefore antibodies. Invertebrates must rely solely on their innate immune response, which is non-specific and is thought to have limited immunological memory.

Control of viral infection in vertebrates typically occurs firstly via the innate branch of the immune system followed by the adaptive branch where memory is established via humoral factors such as antibodies [51]. In the vertebrate system pathogen-associated molecular pattern molecules (PAMPs) such as viral nucleic acid are recognised via pattern recognition receptors (PRRs) [52]. Vertebrates have a discreet range of PRRs that recognise a broad range of viral PAMPs such as double-stranded RNA (dsRNA) and cytosolic DNA [53]. The activation of PRRs is able to trigger a signalling cascade, activating the production of cytokines such as interferons [52,53], which are secreted from infected cells, and initiate an anti-viral state by binding to their associated receptors which are found on all nucleated cells [52]. This activates the Janus kinase/signal transducers and activators of transcription (JAK-STAT) pathway resulting in the transcription of hundreds of interferon-stimulated genes (ISGs) [54]. ISGs have various anti-viral roles [48,52] and can inhibit viral pathogenesis by directly disrupting essential viral pathways and functions needed for the completion of viral lifecycles; as such, interferons and their downstream ISGs are integral to the anti-viral immune response of vertebrates [52].

**Figure 1:**
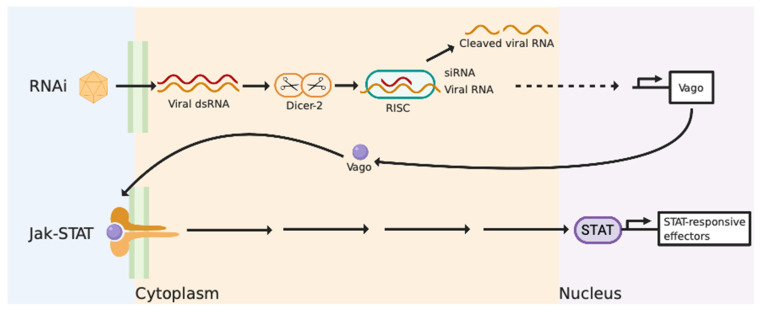
A schematic representation of the relationship between the RNAi and Janus kinase/signal transducers and activators of transcription (JAK-STAT) pathway in invertebrates. The RNAi pathway induces the degradation of viral RNA sequences via pre-RNA-induced silencing complex (RISC). The upregulation of Vago via Dicer-2 initiates the JAK-STAT pathway resulting in the transcription of anti-viral effectors. Diagram created with BioRender.com.

Most of the knowledge on the anti-viral immunity of invertebrates is based on model organisms such as Fruit flies (*Drosophila*) and mosquitos (*Anopheles*). Previous research on these model species has established that they do not elicit an interferon-like response due to the absence of homologous IFN and many ISGs in their genomic sequences [48,55]. Instead, RNA interference (RNAi) is recognised as a significant [48,56,57] and robust anti-viral pathway in these organisms, particularly in insects and nematodes [58]. Virally derived dsRNA is exploited as a template to produce small interfering RNAs (siRNAs) via Dicer-2, as shown in Figure 1 [55,58]. These siRNAs function to bind to complementary viral RNA sequences via the pre-RNA-induced silencing complex (RISC) to disrupt and degrade them, ultimately inhibiting viral replication [55,58]. A transcriptional response to viral infection in arthropods is present but it is dependent upon Dicer-2 recognising dsRNA in an RNAi independent mechanism [48,59]. Upon recognition of dsRNA, Dicer-2 induces the expression of Vago, a secreted peptide that activates the JAK-STAT pathway and initiates the expression of antiviral effectors in a similar fashion to the vertebrate pathway (Figure 1) [48,58]. It is important to note here that although such approaches of using model invertebrate species have characterised important aspects of invertebrate anti-viral immunity, they have failed to address the complexity of other invertebrate systems such as the molluscan anti-viral response.

The ocean environment is exceptionally rich in microbes, and filter and grazing feeders such as oysters and abalone are highly exposed to an array of potential pathogens [60,61]. It can therefore be inferred that these organisms must have a well-equipped innate immune system to not only resist infections, but to simply survive in these environments [60]. It is now well established that molluscs have a complex anti-viral response that is comprised of several immune pathways, effectors and adaptors based on observations in the pacific oyster (*C. gigas*) transcriptome [62].

Research into the *C. gigas* genes that are expressed during viral challenge have inferred that an interferon-like pathway exists in these organisms, despite their genomes not encoding a protein that is homologous to vertebrate interferon [48]. Analysis of the *C. gigas* transcriptome has led to this assumption by way of the discovery of homologs of several PRRs involved in anti-viral signaling, including RIG-like-receptors (RLRs) and Toll-like-receptors (TLRs) [63,64,65]. Moreover, recent evidence suggests that oyster haemolymph contains a heat-stable, protease-susceptible factor that induces the transcription of multiple ISGs in haemocytes, including viperin, a highly conserved and important protein in inhibiting viruses [54,63]. The genome of *C. gigas* encodes a diverse set of 83 TLRs, which is incredibly extensive when compared to the 9 and 10 TLRs encoded by fruit flies and humans, respectively [62]. It is thought that the diverse and expanded set of TLRs compensates for the lack of an adaptive immune system and is especially useful to recognise complex pathogens [60]. Homologs also exist for several other factors, including the members of the JAK-STAT pathway, stimulator of interferon genes (STING) and interferon regulatory factors (IRFs), which are all key players in the vertebrate anti-viral innate system [63].

In the early stages of OsHV-1 infection, *C. gigas* adults and juveniles mount an anti-viral response constituting of apoptosis, virus recognition, immune signalling and upregulation of known antiviral effector genes [48]. Of these, the antiviral effectors of particular interest are viperin, double-stranded RNA-specific adenosine deaminase (ADAR-L), IFI44 and protein kinase R (PKR) because they are upregulated in the presence of OsHV-1 [48,65]. In vertebrate models, these effectors are known to have an important role in inhibiting viral replication by way of targeting transcription and translation of both host and viral proteins, however, their anti-viral role is yet to be fully established in invertebrates [48,52,66]. Of particular interest is the potential of exploiting ADAR-mediated editing via ADAR-1 as an anti-viral control against Malacoherpesviruses [67]. ADAR-1 was recently reported to be present in both *C. gigas* and *Haliotis diversicolor supertexta* infected with OsHV-1 and HaHV-1, respectively [67]. It has been demonstrated that in hybrid abalone (*Haliotis laevigata* and *Haliotis rubra*) circulating haemocytes play a role in the initial stage of infection with HaHV-1 [68]. In general, haemocytes are known to clear microbial infections through phagocytosis and release compounds that induce cytotoxic reactions [69]. It has also been assumed that apoptosis, which is a form of programmed cell death, may play a critical role in the immune response of molluscs [39,48]. In its genome, OsHV-1 encodes four inhibitors of apoptosis, encoded by ORFs 42, 87, 99 and 106, which are highly expressed during the stages of early infection [48,54,70]. Vertebrate herpesviruses are known to modulate host immune responses for successful dissemination so HaHV-1 could also potentially have these properties [39,54].

To further emphasise the complexity of the molluscan immune response, a study which compared the anti-viral and anti-bacterial immune responses of juvenile and adult *C. gigas* found that immune responses are tailored depending on the type of pathogen, implying that the oyster can distinguish between a viral and a bacterial infection, and respond differentially, perhaps giving us an indication that they harbor a diverse range of functional pattern recognition receptors. [71]. Interestingly, this study and others have demonstrated that juvenile oysters are more susceptible to OsHV-1, however they mount an effective anti-bacterial response to *Vibrio spp*., perhaps further highlighting the complexity of their immune system [16,71,72]. The increased susceptibility of juvenile oysters following initial field studies was hypothesized to be due to an immature immune system incapable of mounting an effective response to OsHV-1 [12]. However, it is now understood that juveniles actually mount an ‘over the top’ and perhaps unregulated immune response, which may create a toxic cellular environment which ultimately results in their death [64,71]. It is clear that our understanding of the immune responses of both oysters and abalone are in their infancy, with more studies having been done in the oyster; however, an improved understanding of the anti-viral immune responses in these organisms and its complexity will likely assist in providing a foundation into understanding potential control mechanisms against OsHV-1 and HaHV-1

## 4. Immune Based Strategies for Control of Herpesvirus in Molluscs

The current means of controlling viral spread in aquaculture are inadequate and this was mirrored through the mass mortality events during outbreaks of OsHV-1 in France and HaHV-1 in Australia [16,19,73]. Although the genetic resistance profile of oysters and abalone species to OsHV-1 and HaHV-1 is not well understood, there are current efforts in their infancy to selectively breed OsHV-1 pacific oysters worldwide, which has had mixed results [74,75,76,77]. However, the current means of viral control still include biosecurity methods, such as quarantine, movement restrictions and destruction of diseased stock, which would generally be effective in an agricultural setting [18]. Unfortunately, such measures are difficult and impractical to enforce in molluscan farming systems as the water housing the animals is generally pumped directly from the ocean [18]. Moreover, these practices are only employed once clinical signs arise so it can be assumed that these animals have already shed virus into the environment, indicating that such practices are inadequate in controlling spread. This highlights the need for an alternate strategy for use in such conditions.

The discovery that organisms lacking an adaptive immune system possess the ability to implement an enhanced immune response upon second exposure indicates some form of innate immune memory [78,79]. As such, the distinct boundaries surrounding the characterisation of the innate and adaptive immune response should be revised [78,80]. This “memory” is broadly defined as immune priming and it has been theorised to occur in one of two ways. One way could mirror the immune memory of vertebrates where a specific pathogen is recognised upon second exposure [63,81]. The immune system is thought to emit a large response clearing the pathogen in a faster and more direct manner than the first time [81,82]. The other involves a non-specific immune response that is sustained at high levels after the first response despite complete neutralisation of the pathogen [81,82]. It has recently been elucidated that poly(I:C) primed *C.gigas* are most likely to behave in a way similar to the latter, based on the sustained expression profile observed in the transcriptome of these animals [83]. Although, when looked at from a vertebrate point of view, some may argue that this does not specifically confer as immune memory but it still implies that pre-exposed invertebrates harbour an immune advantage over naïve animals [57]. This phenomenon has been termed immune priming and is known to offer protection from pathogens including bacteria, parasites and viruses in several organisms [57].

Immune priming has been explored in a range of organisms, such as the penaeid shrimp and more recently in the mollusc, *C. gigas*. Shrimp are susceptible to white spot syndrome virus (WSSV), the etiological agent of white spot disease, but it has been shown that immune priming these organisms with viral components such as envelope glycoproteins offers protection from doses of WSSV that would normally be lethal [84]. It has been hypothesised that multiple methods could explain this phenomenon in penaeid shrimp [84]. These include a specific haemocyte response comparable to vertebrate antibodies and a cell-specific response that involves the blocking of receptors essential for viral attachment and entry, however these mechanisms remain unconfirmed, emphasising the need for more research in this area [84,85]. Literature has more recently explored the phenomenon of immune priming in *C. gigas* and the basis of these studies forms around the injection of poly(I:C) which is an immune-stimulant consisting of non-infectious dsRNA [56]. Poly(I:C) has the ability to mimic viral infection because during replication, dsRNA is produced as an intermediate by DNA viruses such as herpesviruses [66,78,86]. It is recognised in the literature that poly(I:C) of differing lengths is able to elicit an anti-viral response in *C. gigas* whereby a large proportion of primed animals can resist OsHV-1 infection for up to five months following immune priming [56,78]. The mechanisms and route of protection following immune priming with poly(I:C) is not completely understood, although it has been demonstrated that the immune stimulant upregulates a TLR, an IRF and an NF-κB response, therefore stimulating an anti-viral state [56,86]. Although it has been shown that immune priming is effective in protecting *C. gigas* from OsHV-1, it is not feasible to immune prime individual animals, especially in an aquacultural setting where farms likely contain millions of animals. This is where trans-generational immune priming may become advantageous [6].

Broadly defined, trans-generational immune priming (TGIP) is the phenomenon by which a parent can transfer immunological information based on previous infections in the form of protection to its offspring [87]. Such a method is favourable in molluscan farming systems since abalone and oysters are highly fertile, which is a trait exploited by farms through the generation of copious amounts of offspring from a small group of parents [6]. In an earlier study, it was concluded that the susceptibility of parent *C. gigas* to OsHV-1 had an influence on the susceptibility and rates of survival in progeny [88]. Based on their results, the study hypothesised that parents infected with OsHV-1 have the ability to pass on some form of anti-viral protection to their offspring [88]. This is particularly interesting since invertebrates lack antibodies which are known to be the main form of maternal immunity passed on to offspring in vertebrate systems [87]. Subsequent studies have also shown that immune priming oyster parents with poly(I:C) also affords progeny with double the chance of survival to OsHV-1 infection when compared to un-primed control parents [6]. Male parents have since been found to be less effective in these TGIP studies [57,89]. As with immune priming, the mechanisms of the phenomena of TGIP remain unknown. One potential explanation could involve epigenetic mechanisms which are heritable changes in gene expression without directly altering the DNA sequence itself [63]. Examples of such are DNA methylation or non-coding RNA which have the ability to heighten constitutive expression of anti-viral effector genes [61], however more work needs to be done in this field to fully elucidate TGIP mechanisms. Although the mechanisms remain unknown, recent research highlights the potential of implementing TGIP in large scale molluscan farming to offset losses caused by disease; but caution should be taken as it is also not known if trade-offs will occur in desirable production traits for enhanced immunity.

## 5. Future Directions for Control of Herpesvirus in Molluscs

As discussed, research surrounding molluscan herpesviruses, OsHV-1 and in particular HaHV-1 is in its infancy. An abundance of literature explores immunity in *C. gigas* which has given a broader perspective to the complexity of the invertebrate immune system and potential anti-viral control strategies. Despite these advances, research regarding the anti-viral response in abalone is still fragmentary when we consider the importance of this mollusc in commercial farmed aquaculture. As discussed in this review, the invertebrate immune response differs between classes of organisms so it cannot be assumed that abalone will have the same or similar immune response to herpesviruses in the same manner as oysters. It also remains unknown whether research investigating both genetic breeding programs or immune priming strategies and TGIP will likewise protect abalone against HaHV-1 as is seen for infection of oysters with OsHV-1.

Currently immune priming via injection of poly(I:C) is not a viable control strategy in molluscs due to the volume of animals in aquacultural settings. This highlights the advantageous qualities of TGIP although it is unclear if potential trade-offs with desirable production traits such as growth rates could occur [6,87,90]. A feed based immune primer could be implemented in farmed environments, although it is important to note that several variables have the ability to affect the potential of TGIP. It has been shown in other invertebrates, such as the round worm and fruit fly, that the route of parental priming (injection vs. ingestion), as well as the type and form of the immune primer (inactivated vs. living pathogen) can also affect how transmissible the protection is [87]. Moreover, in spite of the advances in our knowledge based on oysters, many gaps still exist in our understanding of these bivalves and more so for the gastropod abalone. Future research efforts focusing on filling such gaps in our knowledge are required to generate practical solutions with the potential to control molluscan herpesviruses in aquaculture.
